# Effects of dietary nitrate on respiratory physiology at high altitude - Results from the Xtreme Alps study

**DOI:** 10.1016/j.niox.2017.10.005

**Published:** 2017-12-01

**Authors:** Andrew F. Cumpstey, Philip J. Hennis, Edward T. Gilbert-Kawai, Bernadette O. Fernandez, Matthieu Poudevigne, Alexandra Cobb, Paula Meale, Kay Mitchell, Helen Moyses, Helmut Pöhnl, Monty G. Mythen, Michael P.W. Grocott, Martin Feelisch, Daniel S. Martin, for the Xtreme Alps research group

**Affiliations:** a Critical Care Research Area, Southampton NIHR Respiratory Biomedical Research Unit, Tremona Road, Southampton, SO16 6YD UK; b Anaesthesia and Critical Care Research Unit, University Hospital Southampton NHS Foundation Trust, Tremona Road, Southampton, SO16 6YD UK; c Integrative Physiology and Critical Illness Group, Clinical and Experimental Sciences, University of Southampton, Tremona Road, Southampton, SO16 6YD UK; d UCL Centre for Altitude, Space and Extreme Environment (CASE) Medicine, UCLH NIHR Biomedical Research Centre, Institute of Sport Exercise & Health, 170 Tottenham Court Road, London, W1T 7HA, UK; e Clinical & Experimental Sciences, Faculty of Medicine, NIHR Southampton Biomedical Research Centre, University of Southampton and University Hospital Southampton NHS Foundation Trust, Tremona Road, Southampton, SO16 6YD UK; f Warwick Medical School, Division of Metabolic and Vascular Health, University of Warwick, Gibbet Hill Road, Coventry CV4 7AL, UK; g AURAPA, Paul-Heidelbauer-Straße 26, 74321 Bietigheim-Bissingen, Germany

**Keywords:** Nitrate, Nitrite, Altitude, Nitric oxide, Hypoxia, Hypoxaemia

## Abstract

Nitric oxide (NO) production plays a central role in conferring tolerance to hypoxia. Tibetan highlanders, successful high-altitude dwellers for millennia, have higher circulating nitrate and exhaled NO (E_NO_) levels than native lowlanders. Since nitrate itself can reduce the oxygen cost of exercise in normoxia it may confer additional benefits at high altitude. *Xtreme Alps* was a double-blinded randomised placebo-controlled trial to investigate how dietary nitrate supplementation affects physiological responses to hypoxia in 28 healthy adult volunteers resident at 4559 m for 1 week; 14 receiving a beetroot-based high-nitrate supplement and 14 receiving a low-nitrate ‘placebo’ of matching appearance/taste. E_NO_, vital signs and acute mountain sickness (AMS) severity were recorded at sea level (SL) and daily at altitude. Moreover, standard spirometric values were recorded, and saliva and exhaled breath condensate (EBC) collected. There was no significant difference in resting cardiorespiratory variables, peripheral oxygen saturation or AMS score with nitrate supplementation at SL or altitude. Median E_NO_ levels increased from 1.5/3.0  mPa at SL, to 3.5/7.4 mPa after 5 days at altitude (D5) in the low and high-nitrate groups, respectively (p = 0.02). EBC nitrite also rose significantly with dietary nitrate (p = 0.004), 1.7–5.1  μM at SL and 1.6–6.3 μM at D5, and this rise appeared to be associated with increased levels of E_NO_. However, no significant changes occurred to levels of EBC nitrate or nitrosation products (RXNO). Median salivary nitrite/nitrate concentrations increased from 56.5/786 μM to 333/5,194  μM  with nitrate supplementation at SL, and changed to 85.6/641 μM and 341/4,553 μM on D5. Salivary RXNO rose markedly with treatment at SL from 0.55 μM to 5.70 μM. At D5 placebo salivary RXNO had increased to 1.90 μM whilst treatment RXNO decreased to 3.26 μM. There was no association with changes in any observation variables or AMS score. In conclusion, dietary nitrate supplementation is well tolerated at altitude and significantly increases pulmonary NO availability and both salivary and EBC NO metabolite concentrations. Surprisingly, this is not associated with changes in hemodynamics, oxygen saturation or AMS development.

## Highlights

• Xtreme Alps is a randomised controlled field study of dietary nitrate at altitude.
• Nitrate increased levels of exhaled NO and salivary nitrite, nitrate & RXNO.
• Breath condensate nitrate, oxygen saturation and blood pressure did not change following dietary nitrate supplementation.
• Increases in exhaled NO correlated with increases in breath condensate nitrite.
• Dietary nitrate did not alter the incidence of Acute Mountain Sickness at 4559 m.

## 1 Introduction

The partial pressure of oxygen decreases on ascent to high altitude as a result of a decline in barometric pressure; at 5300 m (the height of Everest base camp) it is approximately half the value at sea level [1]. This hypobaric hypoxia causes hypoxaemia (a lack of oxygen in the blood) resulting in significant physiological challenges. The respiratory system is particularly affected, and consequently respiratory problems make up some of the most common (e.g. high altitude cough) and also the most serious (e.g. high altitude pulmonary oedema or HAPE) illnesses encountered at high altitude [2]. Respiratory pathologies and hypoxaemia are also commonly encountered in critical care patients but are very difficult to study in this setting. High altitude environments might therefore provide a model for pathological hypoxaemia from which much can be learned [3].

Interestingly, native lowlanders appear to respond to acute hypobaric hypoxia differently from native highlanders (i.e. populations permanently residing above 3000 m). For example, native lowlanders seem to rely heavily on increased erythropoiesis to maintain oxygen content at altitude [1], whilst this response is much less pronounced in native highlanders such as the Sherpas (supremely adapted after living at high altitude for over 500 generations) [4]. Another notable difference that is remarkably conserved across different high altitude populations (including the Tibetan populations and the Bolivian Aymara) is that mountain dwellers exhale higher concentrations of nitric oxide (NO) compared to individuals living at sea level [5], [6]. However, exhaled NO levels decrease significantly in lowland populations on acute exposure to altitude [7]. During graded exposure to altitude, however, NO production increases also in lowlanders suggesting that NO production and particularly respiratory NO availability might play an important role in offsetting the effect of hypoxia and improve performance [8].

NO is a ubiquitous signalling and effector molecule, and an important modulator of oxygen delivery through effects on blood pressure and blood flow as well as mitochondrial respiration. As well as these vasoactive properties, it also plays crucial roles in inflammation (including pulmonary and airway inflammation) and in the immune system, as a cytotoxic entity and by abating oxidative stress [9]. Moreover, in hypoxia it plays a key role in energy supply-demand matching [10]. Inhaled NO has been shown to reduce pulmonary artery pressures and increase arterial oxygenation in critical care patients suffering from Acute Respiratory Distress Syndrome by improving ventilation-perfusion matching [11]. Inhaled NO has also been used to treat patients suffering from HAPE, successfully reducing oedema and increasing arterial oxygenation [12].

NO is produced through two main pathways in the body, both regulated by oxygen. While the enzymatic formation of NO from l-arginine by nitric oxide synthases (NOS) is an oxygen-requiring process, production of NO through serial reduction of inorganic nitrate (NO_3_^−^) to nitrite (NO_2_^−^) and NO is inhibited by oxygen [13]. It is this second mechanism that is thought to underlie the significant increase in markers of NO metabolism observed in acclimatizing lowlanders and also well-adapted Sherpas at high altitude [8]. Dietary nitrate is initially reduced to nitrite by the oral bacterial flora in the mouth, before nitrite can be further reduced to NO in the acidic environment of the stomach and in vascular tissue by various nitrite reductase enzymes [14], [15], [16].

Besides its use as an ergogenic aid in sports medicine [17], [18], [19], [20], [21], attempts to increase nitrate intake through dietary supplements have also been trialled as therapeutic interventions in respiratory patients suffering from chronic obstructive pulmonary disease (COPD), which results in a chronic hypoxaemic state, with mixed results [22], [23], [24], [25], [26]. Some studies showed beneficial effects such as increased exercise performance and lower resting blood pressure [22], [24], whilst other studies did not show any significant improvements in exercise performance [25], [26]. Some studies only showed limited improvements (such as reduced oxygen consumption) but suggested that these benefits might be greatest in the most hypoxic patients [23]. However, all of these studies demonstrated that dietary nitrate supplementation is both safe and tolerable to patients. If dietary nitrate supplementation could improve physiological performance under other hypoxic conditions then it could potentially be an effective and economic treatment for hypoxaemia in critically ill patients.

The Xtreme Alps expedition was a double-blinded randomised placebo-controlled trial designed to investigate how dietary nitrate supplementation affects performance at altitude [27]. This report focuses on the findings of the study's respiratory outcomes, baseline observations and acute mountain sickness (AMS) scores.

## 2 Methods

### 2.1 Study setting and participants

Details of the Xtreme Alps expedition and protocols have been previously described [27]. A total of 28 healthy adult volunteers (aged 21–40 years) were recruited after completing a health screening process to identify those at risk of problems at high altitude (as detailed in [27]). Of these, 21 (75%) were male. The mean weight was 73.3 kg (±11.6), mean height 1.76 m (±0.08), and mean BMI 23.6 (±2.7). 3.6% were smokers and 75% had previously been to an altitude of over 3000 m. Anyone deemed unfit to undergo a formal exercise test at high altitude (as previously described [28]) was excluded. Written informed consent was obtained from each participant and ethical approval for the study was obtained from Research Ethics Committees at both University College London, UK and the University of Turin, Italy.

**Fig. 1:**
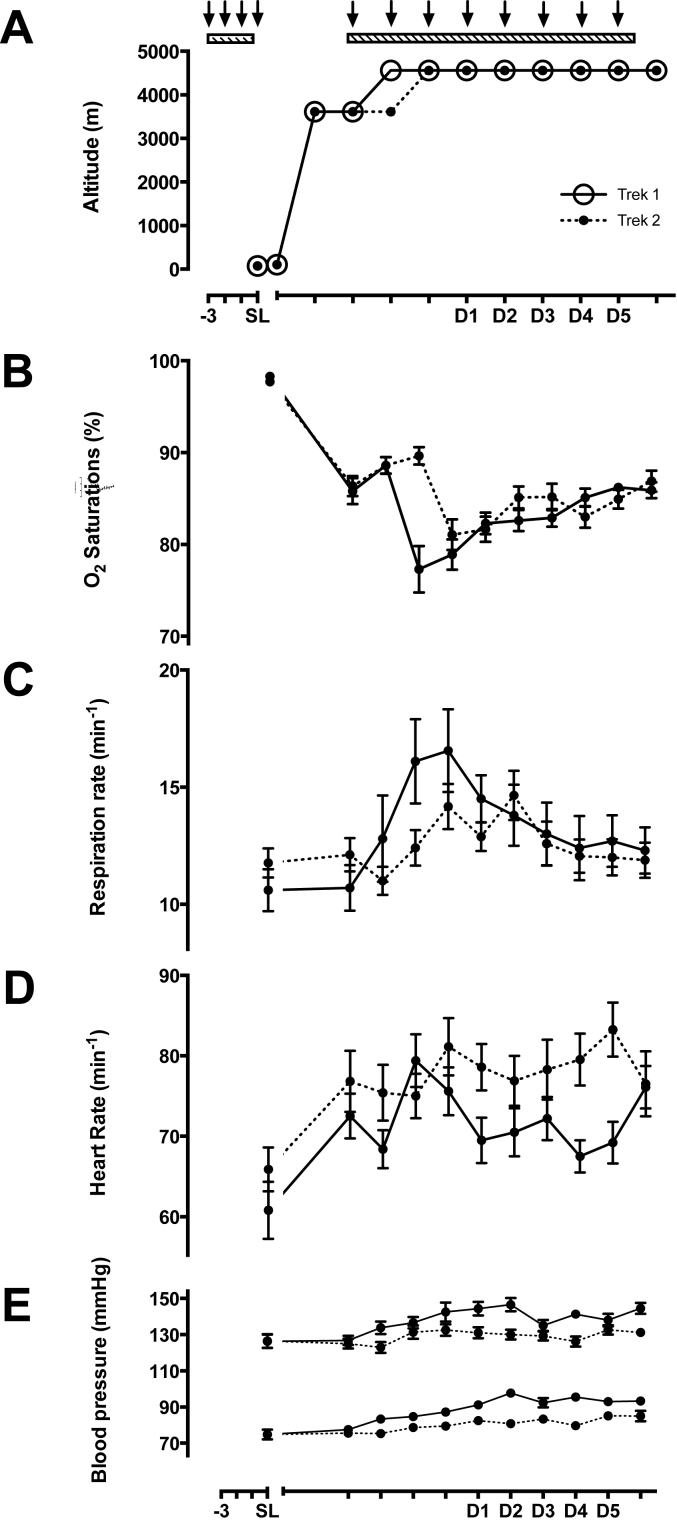
Changes in resting physiological variables during the Xtreme Alps expedition. All values are presented as means ± SEM, displayed separately for Trek 1 and Trek 2 (solid and dotted lines, respectively) as Trek 1 ascended one day faster from the Gnifetti Hut (3611 m) to the Margherita Hut (4559 m) due to inclement weather conditions. Each arrow represents the administration of 3 x doses of either treatment or placebo supplement (1 dose before every meal each day), starting 3 days prior to each testing period. SL = sea level testing, D1 – D5 = altitude testing days 1–5. Panel 1A - ascent profile, 1B - resting oxygen saturation per expedition day, 1C – resting respiration rate, 1D – resting heart rate, and 1E – resting blood pressure (systolic over diastolic).

The study took place in August 2010 at the Capanna Regina Margherita (‘Margherita Hut’) on the summit of Monte Rosa (altitude 4559 m), which contains a research laboratory inside, managed by the University of Turin. Baseline measurements were conducted in London (altitude 75 m) six weeks earlier: mean barometric pressure 100.5 kPa, mean laboratory temperature 24.1 °C and mean oxygen partial pressure 19.7 kPa. Participants were then separated into 2 groups (Trek 1 and 2) based on their availability, with Trek 2 starting their ascent seven days after Trek 1. All investigators and participants initially flew to Milan (102 m) and remained there one night before starting to ascend the following day; by road to Alagna (1205 m), by lift to Punta Indren (3250 m), and then on foot to the Gnifetti Hut (3611 m). Severe weather conditions meant Trek 1 only stayed at 3611 m for two nights before ascending on foot to the Margherita Hut, whilst Trek 2 ascended on foot after three nights at 3611 m. Subjects then remained at 4559 m for the rest of the study duration (eight nights for Trek 1, seven for Trek 2) (see Fig. 1A) before descending on foot again. This schedule included a planned rest day on arrival at 4559 m for each group (during which the first group could build and set up the laboratory), followed by 5 days of testing. In the laboratory at 4559 m, the mean barometric pressure, temperature and partial pressure of oxygen were 78.1 kPa, 22.6 °C and 15.1 kPa respectively.

### 2.2 Randomisation

Following enrolment, participants were randomly allocated to receive either a high nitrate supplement (intervention group) or low nitrate supplement (placebo group) in accordance with the CONSORT 2010 guidelines (http://www.consort-statement. org/) and accounting for the need for equal numbers of individuals in both groups whilst maintaining gender distribution. Both participants and investigators were blinded to group allocations for the duration of the study, including the analysis of the primary data sets.

### 2.3 Intervention

The intervention juice was a custom formulated all-natural beetroot/fruit juice blend (produced, pasteurised and packaged into 200 mL food-grade aluminum foil pouches individually labelled with anonymized subject codes by Aurapa GmbH, Bietigheim-Bissingen, Germany). The total daily nitrate dose targeted by the high nitrate intervention was similar to that shown by others to be effective in improving exercise performance at sea level (range 0.10–0.18 mmol/kg/day) [29], [30], and divided into three individual doses for consumption in the morning, at midday and in the evening. The low-nitrate (placebo) version was produced in the same manner using beetroot juice in which the majority of nitrate had been removed by selective microbial denitrification. Administration, both at sea level and high altitude, commenced 72 h prior to the start of and continued throughout each testing period. However, as all subjects in both the placebo and treatment groups were taking beetroot juice for 3 days prior to each experimental period starting, we were unable to measure baseline (or 'treatment-free' control) measurements either at sea level or at altitude in either of these groups. A limited amount of data is available for E_NO_ and breath condensate levels from treatment-naïve members of the laboratory team, who did not receive any supplement as they were not part of the main study; this data is presented where available on the appropriate figures. However, the error bars for this group are large as the n-values are small and often variable (not all individuals were always available and some unwell, requiring medication and/or experiencing respiratory inflammation on certain days) – these individuals were never intended to be part of the main study but presented here for comparison to the placebo group. All meals consumed by subjects during the study period were standardised in order to minimise nutritional nitrate intake.

### 2.4 Daily diary, measuring observations & AMS scores

All participants completed a daily diary first thing each morning before any oral intake (including food, caffeine, placebo or intervention supplements). Peripheral oxygen saturation (SpO_2_), heart rate (HR), respiratory rate (RR) and blood pressure (BP) were measured after five minutes of rest, sitting upright. Finally, each participant also recorded medications taken and their symptoms according to the Lake Louise score – a validated score for diagnosing and grading AMS [31], [32].

### 2.5 Respiratory measurements

Standard spirometric variables (FEV1, FVC, FEV1/FVC, PEF and FEF 25–75) were measured using an ultrasonic spirometer (Easy-One, NDD Medical Technologies, MA, USA). Due to a corrupted file all spirometric recordings taken at high altitude were lost; therefore, only sea level data are reported.

Breath-to-breath analysis of inhaled and exhaled NO was performed using a spirometric analysis device (using individual disposable Spirettes and bacterial filters) connected to a gas phase chemiluminescence analyzer (CLD88sp with Spiroware software, EcoMedics, Duernten, Switzerland); two identical units were used in parallel throughout. In accordance with current American Thoracic Society/European Respiratory Society recommendations for exhaled NO measurements [33], individual Denox 88 units (EcoMedics, Duernten, Switzerland) provided NO-free air and enabled advanced adaptive expiratory flow control. Expiratory gas flow was measured in real time according to the time-of-flight principle using a high resolution/high frequency ultrasonic device. A fraction of inspired and expired air (∼10% of the expired air at a nominal flow of 50 ml/s) was continually transferred from a sidestream opening in the handheld spirometer; here, flow was controlled using a fixed orifice in the sample line with a vacuum pump driven internal resistor arrangement, maintaining a constant flow of 300 ml/min of sampled air into the analyzer, independent of changes in barometric pressure.

Both chemiluminescence analysers were equipped with additional stainless-steel bulkhead fittings, which supplied their ozone generators with pure oxygen (instead of environmental air) from a cylinder. This hardware adjustment ensured adequate ozone generation to maintain optimal performance even at lower barometric pressures. For consistency, medical grade oxygen was also used to supply the ozone generators at sea level. All measurement devices were calibrated daily, both at sea level and at altitude, with NO (4.00 ppm) using 100% nitrogen as zero gas and a calibrated 100 mL volumetric syringe. The CLD88sp devices measure continuously ambient temperature and pressure by internal sensors. Each morning accurate barometric pressure and temperature were entered into the software for verification purposes as part of the calibration procedure. The CLD88sp takes the ambient pressure into account during the gas calibration and compensates automatically for diurnal pressure changes. Thus, displayed Fe_NO_ concentrations are always accurate and do not require correction for altitude. However, criticism has been raised in relation to other devices and measurement principles using e.g. electrochemical cells for NO detection where readings can vary with changes in partial pressure [34], [35]. Moreover, the partial pressure of metabolically active gases within a body cavity may more accurately represent biological activity [34]. Therefore we here report both fractional exhaled NO (Fe_NO_) and partial pressures of exhaled NO (Pe_NO_).

For each measurement subjects inspired to total lung capacity before slowly expiring against a defined resistance to ensure proper velum closure. During expiration an electronic visual display helped each subject maintain a steady flow of 50 ml/s in accordance with ATS guidelines - a smiley face against a green background was displayed when the ultrasonically measured flow was within limits (50 ml/s ±5%), with red warning signs flashing when the flow fell below the minimum or exceeded the maximum level. A minimum of 3 successful repeats with stable flow rates for at least 6 s were collected for each measurement.

### 2.6 Measures of nitric oxide/nitrate metabolites

Samples of unstimulated saliva were collected using dedicated collection devices (Salimetrics Oral Swabs; State College, PA, USA) immediately before participants ate their midday meal. Swabs were placed sublingually for 2 min at sea level or 5 min at altitude (to enable sufficient volume to be collected for analysis) before collection into a sterile container and centrifugation at 3000 rpm for 15 min; 1 ml of saliva was aliquoted and stored in two separate cryovials at −40 °C for later determination of nitrosation products, nitrite and nitrate. Subjects had not consumed their respective supplement, brushed their teeth, or had recent acute hypoxic or hyperoxic exposure (e.g. as part of other research protocols) before sample collection.

Exhaled breath condensate (EBC), a readout of alveolar surface chemistry, was collected non-invasively by breathing through a mouthpiece connected to a sampling tube (pre-rinsed with ultrapure Millipore water (EMD MilliporeSigma, MA, USA) and dried before use) contained within an insulated pre-cooled aluminium sleeve (RTube^®^, Respiratory Research, Inc., Charlottesville, VA, USA) for 15 min. Two 1 ml EBC aliquots were frozen and stored at −40 °C straight after collection and analysed later in the UK.

Placebo and intervention juices were saved along with representative food samples served at Margherita Hut. Food samples were collected, homogenized in phosphate buffer (or drinking water), frozen at −40 °C straight after homogenisation and analysed later in the UK.

NO metabolite concentrations were quantified using reductive gas-phase chemiluminescence (for determination of total nitrosation products, RXNO) and HPLC (for nitrite and nitrate), as described elsewhere [8].

### 2.7 Analysis plan

Sea level spirometry values were normally distributed when tested. Separate t-tests, performed in GraphPad Prism 6 software (www.graphpad.com/scientific-software/prism), were used to analyse these variables (FEV1, FVC, FEV1/FVC, PEF & FEF 25–75) as there was no requirement to model for different ascent profiles.

Statistical analyses for E_NO_, breath condensate and saliva measurements were all performed using linear mixed modeling in STATA 11 (http://www.stata.com) to account for the multiple time points at which measurements were taken as well as the nitrate intervention. All physiological observations were normally distributed, as were all other measured variables after logarithmic transformation. All statistical tests (except those spirometry values listed above) were performed on parametric log-transformed data. Significance was assumed when p < 0.05. A sensitivity analysis was performed with missing values replaced by mean values to verify the model. Correlation between breath condensate nitrite and E_NO_ was performed on non-parametric untransformed data using Spearman's Rank with a 2-tailed significance test. Physiological observations and AMS scores are reported as means (±standard deviation). For easier reading, all other values are reported in the text as median (interquartile range) of anti-logged/untransformed data (non-parametric) to allow presentation in original units.

## 3 Results

### 3.1 Dietary nitrate intake and supplementation

The nitrate concentration of the placebo supplement was 1.4 (±0.1) mM; consumption of 3 × 200 mL therefore amounted to 11.5 μmoles/kg bw per day (bw = body weight), which is less than the average UK daily intake of around 24.5 μmoles/kg bw per person per day [36]. The intervention supplement had a nitrate concentration of 18.5 (±2.0) mM, translating into >10 times higher dietary nitrate supplementation levels in the intervention group. Nitrite concentration was below 1 μM in placebo and 2.4 μM in the intervention group, which amount to <0.01 and 0.02 μmole/kg bw per day, respectively – both well below the average UK daily intake of 0.53 μmole/kg bw per day. Where available, the limited measurements we have of untreated adults at altitude closely matched the respective values for the placebo group.

Food provided at altitude varied somewhat from day to day, therefore representative food samples were collected from typical meals (breakfast, lunch, dinner). Average ranges per meal for nitrate were as follows: 50–217 μmoles for breakfast, 132–447 μmoles for lunch, and 559–1201 μmoles for dinner. The average ranges per meal for nitrite were considerably smaller, amounting to 0.17–0.46 μmoles for breakfast, 0.64–1.40 μmoles for lunch, and 0.71–2.10 μmoles for dinner. This amounted to an average intake of 18 (±11) μmoles nitrate/kg bw per day and 0.038 (±0.023) μmoles nitrite/kg bw per day, well within normal UK daily intakes [36]. Combined, the placebo group received an average of 29.5 μmoles nitrate/kg bw per day, slightly (20%) above the average UK intake whilst the intervention group received up to 186 μmol nitrate/kg bw per day, or 6.6 times larger than the normal UK daily intake with minimal addition of nitrite from either placebo or intervention to the diet.

### 3.2 Baseline physiological observations

Increasing exposure to hypobaric hypoxia at high altitude produced marked changes in participants' resting physiological variables (see Fig. 1). Resting SpO_2_ initially decreased with each incremental rise in altitude, before stabilising as further time was spent at each elevation (see Fig. 1B). Meanwhile, resting respiration rates (RR) initially increased on ascent before trending back towards baseline values (see Fig. 1C). Mean resting SpO_2_ at sea level was 98.3% (±1.1) and 97.7% (±1.5) for Trek 1 and 2 respectively, decreasing to 77.3% (±8.0) and 81.1% (±6.84) on their first day at 4,559 m (day 3 and day 4, respectively). Mean RRs for Trek 1 and 2 respectively were 10.6 (±2.9) and 11.8 (±2.6) at sea level, increasing to 16.1 (±5.7) and 14.2 (±4.0) at arriving at 4,559 m, and decreasing to 12.3 (±3.1) and 11.9 (±3.1) by day 10. A similar response pattern was also seen for resting heart rate and blood pressure (Fig. 1D and E). All physiological trends were similar between both ascent profiles; hence all other measurements were pooled for subsequent analysis.

### 3.3 Acute mountain sickness (AMS)

**Fig. 2:**
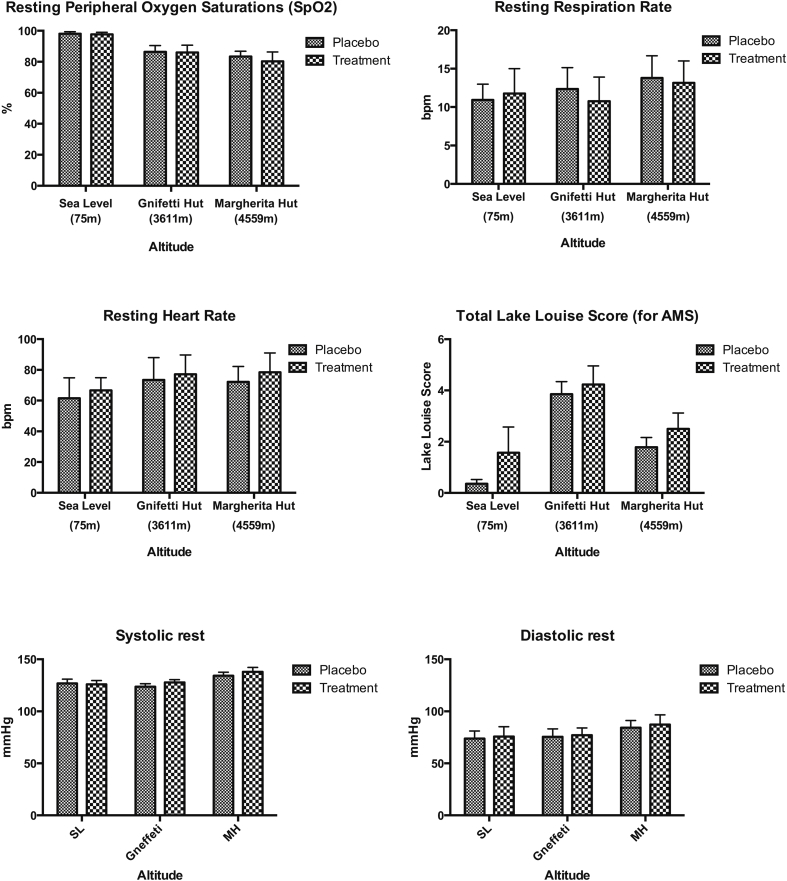
Representative results of the daily diary morning routine recording resting vital signs and symptoms of Acute Mountain Sickness (AMS). No significant difference was seen between the low nitrate group (placebo) and the high nitrate group (intervention) in peripheral oxygen saturation (SpO_2_), respiratory rate, heart rate, systolic blood pressure, diastolic blood pressure or Lake Louise Score (for AMS) on testing at sea level in London (75 m), on participants' first day at 3611 m or on the first testing day at 4559 m (D1 in Fig. 1).

There was no significant difference in the incidence of Acute Mountain Sickness (AMS) between the intervention and placebo groups, p = 0.29 (see Fig. 2). Similarly, the dietary nitrate supplement did not significantly alter the incidence of headache at altitude (p = 0.47). Both of these findings remained consistent when the different ascent rate (different trek groups) was also included in the linear-regression model. One subject was evacuated hours after arriving at 4559 m after developing high altitude cerebral oedema (HACE). No one developed HAPE.

### 3.4 Lung function, exhaled NO and NO metabolite levels in exhaled breath condensate

All sea level spirometry variables were normally distributed. All subjects (2 in the treatment group smoked) had normal lung function and there was no significant difference seen between the placebo and treatment groups for any sea level spirometry variable when tested (see Table 1).

**Table 1:** Summary spirometry data for all subjects at sea level. There is no significant difference between the placebo and treatment groups for any variable measured.

(% pred.)	Placebo	Treatment	P value
FEV1	103.0 ± 3.5	97.6 ± 3.1	0.19
FVC	107.0 ± 2.7	93.0 ± 7.8	0.10
FEV/FVC	99.5 ± 2.3	98.9 ± 1.6	0.83
PEF (l/min)	114.0 ± 3.4	115.0 ± 3.3	0.83
FEV 25-75	92.2 ± 6.1	70.2 ± 13.6	0.15

**Fig. 3:**
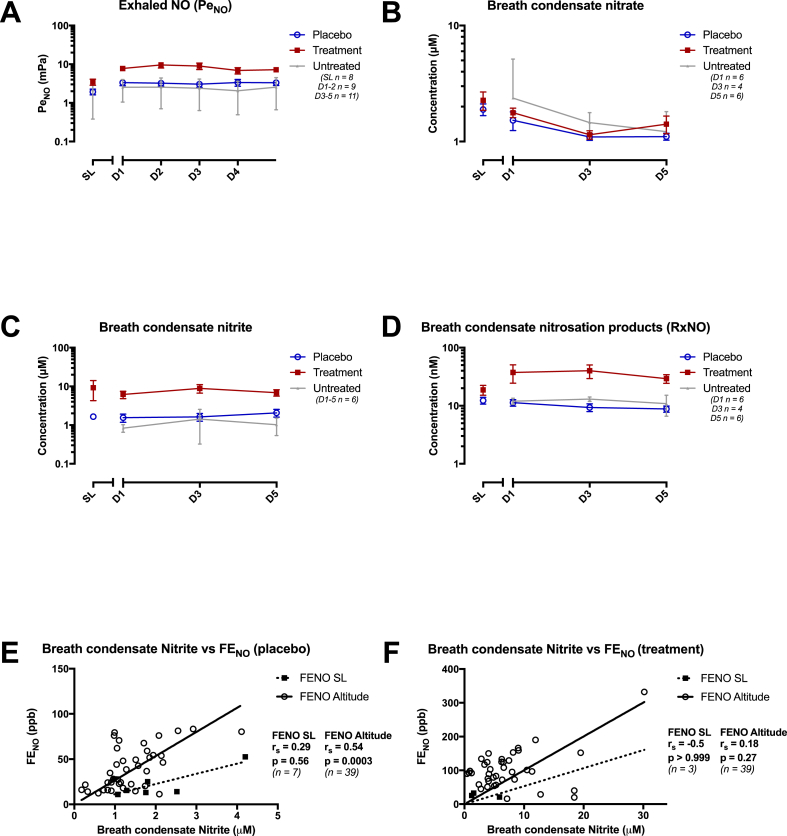
Graphs showing mean exhaled NO and breath condensate values for control (low nitrate placebo) and supplement (high nitrate intervention) groups at sea level (SL) and days 1–5 (D1 - D5) at 4559 m altitude. Where available, corresponding values for untreated laboratory staff members is also presented. Significant rises were seen in exhaled NO and breath condensate nitrite in response to dietary nitrate treatment, but not breath condensate nitrate or RxNO. A: Partial pressure of exhaled NO (Pe_NO_) over time, B: Breath condensate nitrate concentration over time, C: Breath condensate nitrite concentration over time, D: Breath condensate nitrosation products (RxNO) concentration over time. There were moderate correlations between Fractional exhaled NO (Fe_NO_) and Exhaled Breath Condensate nitrite concentrations at sea level and at altitude in both the placebo (graph E) and the treatment (graph F) groups. However, this analysis is limited by low n values at sea level, particularly in the treatment group. r_S_ - correlation coefficient of Spearman Rank test (no adjustment for repeated measures).

All measured breathing and salivary variables were normally distributed after log transformation. As shown in Table 2, Table 4 and Fig. 3 nitrate supplementation and hypobaric exposure both robustly increased E_NO_ levels, and this result remained significant after adjusting for multiple comparisons. Nitrate supplementation also significantly increased nitrite concentrations in EBC.

**Table 2:** Descriptive table for Exhaled NO values at sea level (SL) and 1st (D1), 3rd (D3) and 5th (D5) testing days at 4559 m, expressed as Fractional exhaled NO (Fe_NO_, ppb), and after conversion to partial pressure of exhaled NO (Pe_NO_, mPa). Individual Fe_NO_ measurements were converted to Pe_NO_ measurements using the barometric pressure measured on the specific testing day that each measurement was taken (sea level range: 101.8–102.4 kPa, altitude range: 77.3–79.5 kPa). Data are presented as median (IQR). There was a significant increase in Exhaled NO levels in response to dietary nitrate supplementation in a linear regression model.

	Fe_NO_ (ppb)			Pe_NO_ (mPa)		
	Untreated	Placebo	Treatment	Untreated	Placebo	Treatment
SL	14.0 (11.2–22.8)	14.7 (11.5–25.1)	29.3 (21.0–42.6)	1.43 (1.1–2.7)	1.5 (1.2–2.6)	2.99 (1.98–4.44)
D1	27.7 (20.7–32.7)	36.3 (20.3–70.7)	92.0 (79.0–123.7)	2.2 (1.6–3.7)	2.8 (1.5–5.7)	7.3 (6.1–9.8)
D3	29.0 (11.7–42.6)	32.3 (15.8–53.8)	96.7 (68.3–129.0)	2.2 (0.9–4.0)	2.5 (1.2–4.4)	7.6 (4.9–11.6)
D5	22.8 (18.2–37.0)	43.3 (24.0–54.3)	95.0 (54.3–133.3)	1.8 (1.2–3.1)	3.4 (1.8–4.6)	7.4 (3.9–10.4)

**Table 3:** Descriptive table for sea level (SL) and 1st (D1), 3rd (D3) and 5th (D5) testing days at 4559 m. Data are presented as median (IQR). All variables showed significant increases in response to dietary nitrate supplementation in a linear regression model except for exhaled breath condensate (EBC) nitrate and RxNO.

		Placebo	Treatment
Breath Nitrate (μM)	SL	1.84 (1.42–2.24)	2.20 (1.29–2.90)
	D1	1.07 (0.87–1.74)	1.56 (1.25–2.29)
	D3	1.08 (0.89–1.26)	1.00 (0.92–1.45)
	D5	1.17 (0.93–1.31)	1.20 (1.08–1.39)
Breath Nitrite (μM)	SL	1.66 (1.29–1.80)	5.05 (1.22–9.95)
	D1	1.15 (0.98–1.74)	4.87 (3.94–7.15)
	D3	1.24 (0.91–2.08)	6.60 (4.06–11.35)
	D5	1.64 (1.04–2.09)	6.26 (3.83–8.16)
Breath RxNO (nM)	SL	12.29 (10.02–13.79)	16.15 (11.35–24.53)
	D1	10.02 (8.61–12.74)	20.47 (14.85–41.89)
	D3	8.29 (5.70–11.20)	26.18 (21.67–78.40)
	D5	8.76 (4.69–12.68)	22.61 (18.89–32.81)
Saliva Nitrate (μM)	SL	785.9 (487.7–954.2)	5194.3 (4066.2–5910.6)
	D1	876.1 (499.1–1099.9)	3945.4 (2037.4–6314.8)
	D3	874.5 (516.5–1126.0)	5478.1 (2525.5–12206.1)
	D5	641.3 (474.2–1285.1)	4552.94 (3444.4–5975.4)
Saliva Nitrite (μM)	SL	56.5 (17.1–147.3)	332.8 (133.0–460.2)
	D1	41.7 (17.0–76.6)	170.9 (130.5–680.5)
	D3	40.2 (13.5–84.4)	186.4 (117.5–496.5)
	D5	85.6 (37.3–155.0)	340.9 (282.0–519.4)
Saliva RxNO (nM)	SL	547.9 (340.6–3090.1)	5696.4 (483.8–14897.4)
	D1	681.8 (289.2–1467.6)	4594.9 (632.2–5846.3)
	D3	1260.0 (281.5–2478.8)	4126.22 (2087.3–5448.3)
	D5	1897.50 (462.2–4909.9)	3258.41 (1084.3–7211.9)

**Table 4:** Correlation data for each log variable showing: regression coefficient (β), 95% confidence interval and p value calculated from a linear regression model comparing the effect of the dietary nitrate taking into account the repeated measures at altitude.

Log variable	β	95% Conf. int.	P value
Pe_NO_ (mPa)	0.58	0.087–1.07	0.02
Breath nitrate (μM)	0.21	−0.56–0.98	0.59
Breath nitrite (μM)	1.24	0.41–2.1	0.004
Breath RxNO (μM)	0.42	−0.26–1.10	0.23
Saliva Nitrate (μM)	2.22	1.46–2.98	<0.001
Saliva Nitrite (μM)	2.35	1.21–3.49	<0.001
Saliva RxNO (nM)	1.40	0.062–2.73	0.04

When modeled for days spent at altitude, dietary nitrate supplementation significantly increased E_NO_ levels (p = 0.02), with median values from 1.5 mPA (IQR 1.2–2.6) to 2.99 mPa (IQR 1.98–4.44) at sea level and from 3.4 mPa (IQR 1.8–4.6) to 7.4 mPA (IQR 3.9–10.4) by day 5 at altitude. In the same model, treatment with high nitrate supplementation also significantly increased sea level nitrite concentrations in EBC (p = 0.004), with median values increasing from 1.66 μM (IQR 1.29–1.80) to 5.05 μM (IQR 1.22–9.95) at sea level, and from 1.64 μM (IQR 1.04–2.09) to 6.26 μM (IQR 3.83–8.16) after 5 days at 4559 m. Moreover, association analyses unmasked moderate correlations between measured EBC breath nitrite and E_NO_ levels both at sea level, (r_s_ = 0.29, p = 0.56 & r_s_ = 0.54, p = 0.0003 in the placebo and treatment groups respectively) and at altitude (r_s_ = −0.5, p > 0.999 & r_s_ = 0.18, p = 0.27 respectively). However, this analysis was limited by low n values at sea level, particularly in the treatment group (see Fig. 3). Changes in breath RXNO levels in response to the dietary supplement followed similar trends, but these differences did not reach statistical significance (p = 0.23). No significant change was seen in EBC nitrate concentrations with levels remaining very similar in both treatment groups across the study period (p = 0.59). Median concentrations of nitrate in the low and high nitrate supplements were 1.84 μM (IQR 1.41–2.24) and 2.20 μM (IQR 1.29–2.9) respectively at sea level, and 1.17 μM (IQR 0.93–1.31) and 1.20 μM (IQR 1.08–1.39) after 5 days at altitude.

### 3.5 Oral nitrate reduction and NO metabolite levels in saliva

**Fig. 4:**
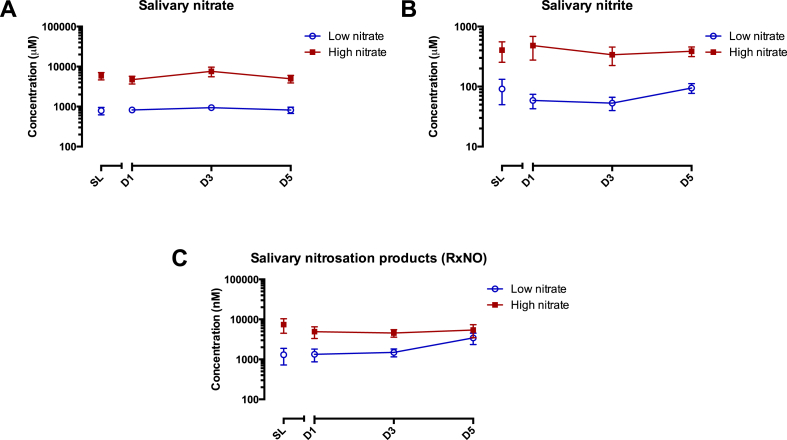
Graphs to values for placebo (low nitrate) and intervention (high nitrate) groups at sea level (day 0) and days 1, 3 & 5 at 4559 m altitude. All values showed significant increases in response to treatment with dietary nitrate. A: Salivary nitrate over time, B: Salivary nitrite over time, C: Salivary RxNO over time.

Similar to EBC, salivary NO metabolite levels at sea level and high altitude varied considerably between individuals. Dietary nitrate supplementation significantly increased salivary levels of nitrate (p < 0.001), nitrite (p < 0.001) and RXNO (p = 0.04) (see Table 3 and Fig. 4). Median salivary nitrate concentrations increased at sea level from 0.79 mM (IQR 0.49–0.95 mM) to 5.19 mM (IQR 4.07–5.91 mM) upon nitrate supplementation, and after 5 days at altitude from 0.64 mM (IQR 0.47–1.29 mM) to 4.55 mM (IQR 3.44–5.98 mM). Median salivary nitrite concentrations also increased with nitrate supplementation, from 56.5 μM (IQR 17.1–147.3) to 332.8 μM (IQR 133.0–460.2) at sea level, and from 85.6 μM (IQR 37.28–155.04) to 340.9 μM (IQR 282.0–519.4) after 5 days at 4559 m.

Initially, similar increases were also seen to median salivary RXNO concentration with the intervention, with levels going from 547.9 nM (IQR 340.6–3090.1) to 5696.4 nM (IQR 483.8–14897.4) at sea level after dietary nitrate supplementation. However the difference between the two randomisation groups was much smaller after 5 days at altitude. The median RXNO concentration in the placebo group increased from sea level to 1897.5 nM (IQR 462.2–4909.9) by day 5, whilst it fell in the supplemented group to 3258.4 nM (IQR 1084.3–7211.9).

A secondary sensitivity analysis did not alter the significance of any of these results. However, despite significant increases in all of these biochemical markers of NO availability, there was no identifiable difference between the intervention and placebo group in any resting physiological variable or score of AMS severity (see Fig. 2).

## 4 Discussion

The novelty of the present study is that it was performed under field conditions (rather than at simulated altitude in a hypobaric chamber) with mountaineer subjects residing at altitude over a prolonged period of time. This experimental design allowed us to take serial measurements in order to examine how dietary nitrate supplementation (ingested in three equal doses throughout each day) affects physiological acclimatization to altitude.

### 4.1 Main findings – exhaled NO (E_NO_)

These results demonstrate that dietary nitrate supplementation with a beetroot/fruit juice mixture at altitude successfully increases nitrate availability; these increases are associated with markedly enhanced levels of exhaled NO (E_NO_), and nitrate and nitrite levels in saliva as well as nitrite concentrations in EBC throughout the duration of altitude exposure. In addition, nitrate supplementation also increased the steady-state concentrations of nitrosation products (S-nitrosothiols and N-nitrosamines) in saliva and compensated for the drop in total nitroso species content in EBC observed following ascent to high altitude. Unexpectedly, despite these significant biochemical changes there was no measureable difference in SpO_2_, RR, BP, HR or AMS severity associated with dietary nitrate supplementation, either at sea level or at high altitude.

Reports on altitude-related changes in E_NO_ of lowlanders and experimental animals are inconsistent. Data from one study in healthy volunteers suggested that E_NO_ levels decrease on acute exposure to altitude, possibly by as much as 33% at a simulated altitude of 5000 m [37]. This was thought to be due to the lower gas density at reduced barometric pressures increasing the back diffusion and alveolar uptake of NO [37], [38]. By contrast, E_NO_ levels increased upon acute nitrate administration in another study [39]. Similarly, E_NO_ increased in rats exposed to 4 days of hypoxia, possibly as an adaptive response to the increased oxidative stress seen with chronic hypoxic exposure [40]. Our findings suggest equivalent responses might also be seen in humans at altitude and over comparable timescales; ascent to 4559 m over at least 3 days demonstrated similar increases in E_NO_ levels in our cohort.

Growing evidence supports the notion that both NO and nitrate metabolism play important roles in the physiological response to hypoxia, and also the benefits that nitrate supplementation can confer on individuals' physiological performance [10], [41]. However, the physiological role of E_NO_ specifically in either of these situations is unclear, and clinically the measurement of Fe_NO_ has mainly been as a point-of-care test for airway inflammation in patients with asthma [42]. Its association with the development of AMS is much less certain.

While it has been suggested that E_NO_ may be associated with AMS, this is not without controversy. One of those studies that led to this conclusion was performed in a hypoxic chamber and subjects only experienced 6 h of exposure to a normobaric hypoxic stimulus [43]. Others suggested that E_NO_ might correlate with the incidence of AMS, but this was a much smaller study (n = 8) where only baseline values for Fe_NO_ were measured and subjects did not receive any dietary intervention [44]. Another study demonstrating that E_NO_ values dropped significantly on acute exposure to high altitude found no association between E_NO_ values and reported symptoms of AMS [7].

The two most striking differences between these studies and the results presented here are our dietary intervention to boost nitrate metabolism and the duration of hypoxic exposure. Our study measured changes over five days at altitude whereas the studies of MacInnis et al. and Brown et al. only looked at an acute exposure to hypoxia, measuring changes up to six hours and three hours, respectively. A recent study that explored a more prolonged exposure to hypobaric exposure where teenage subjects received dietary nitrate supplementation on a graded ascent over two weeks to 5300 m in Nepal also reported no change in AMS scores, lending further support to the unexpected findings of the current study [45].

Conceivably, our nitrate supplement might not have induced a large enough increase in E_NO_ to generate a measurable physiological change in either baseline observations (e.g. SpO_2_) or AMS score. We are not aware of any specific data that would have quantified the increase in E_NO_ required to induce a change in either AMS, SpO_2_ or any other measure reported here in native lowlanders. However, our nitrate supplement successfully doubled median E_NO_ values compared to control so that by day 5 the median E_NO_ in the intervention arm was almost three times higher than levels measured earlier in healthy Tibetan highlanders [46]. Tibetans have less AMS than lowland trekkers even with this accepted upper limit for E_NO_ concentrations; therefore, those supra-normal values in our cohort should have been sufficient to trigger a physiologically meaningful response [47].

### 4.2 Exhaled breath condensate (EBC) results

EBC is being increasingly used to detect levels of airway inflammation, monitor disease progression and also assess disease severity in a number of respiratory conditions including adults and children with atopic asthma [48], [49]. However, despite offering a relatively cheap, quick, simple and non-invasive window into lung physiology, its usefulness in altitude-related respiratory illnesses is less explored.

There is some evidence to suggest EBC could be useful in predicting altitude-related illness. Results from a small study (5 subjects + 11 controls) suggested that EBC analysis before and after 90 min of normobaric hypoxic (12% oxygen) exposure might be able to distinguish between individuals with and without a history of HAPE [50]. Meanwhile, another study demonstrated that EBC measurements conducted at 670 m and 3000 m of malondialdehyde (MDA), a marker of oxidative stress, significantly correlated with the AMS scores of 10 soldiers at 5000 m as they ascended a 6125 m peak [51]. Other oxidative stress markers (e.g. 8-iso prostaglandin F_2α_) have also been shown to significantly increase in EBC measurements in small numbers of both exercising biathletes (n = 10) and sedentary volunteers (n = 5) over a six week period at 2800 m, further supporting our findings presented here [52]. In a study similar to ours also making EBC measurements at 4342 m from seven subjects receiving dietary l-arginine supplementation – an intervention that should increase endogenous NO production via the classical nitric oxide synthase pathway - no improvement in AMS scores were seen with supplementation. In fact, this study found that the treatment group actually developed significantly worse headaches [53].

Again, the biggest difference between all of these previous studies and the results we present here is the scale of the study. To the best of our knowledge, the Xtreme Alps expedition is currently the largest study and 4559 m is also the highest altitude at which EBC samples have yet been collected. While no association was found between NO metabolite related changes in EBC and AMS in the present study, future well-powered studies are needed to determine whether or not this technique could help predict or diagnose altitude illness using other biomarkers.

A curious finding was that nitrite and nitrate concentrations in EBC measured in the present study were almost identical. This is unlike any other compartment where nitrate levels are typically exceeding nitrite levels by at least an order of magnitude. Moreover, we found that dietary nitrate resulted in significant rises to levels of EBC nitrite - with almost identical rises seen both at sea level and at altitude - but almost no difference to EBC nitrate. This strongly suggests that both EBC nitrate and nitrite are under active regulatory control, even when the exact mechanisms of sensing and regulation remain unclear. We also noted moderate associations between levels of EBC nitrite and Fe_NO_, even in the low nitrate/placebo group and particularly at altitude. We believe that, for the first time, this provides evidence to suggest that nitrite in airway secretions might be acting as a possible source of E_NO_. Whether or not these observations are of biological significance warrants further investigation.

### 4.3 Enterosalivary nitrate circulation

The physiological importance of the enterosalivary nitrate circulation has only been realised recently whilst the conversion of l-arginine and oxygen to NO and citrulline by NOS enzymes was established two decades earlier [54], [55]. NOS-independent nitric oxide production was not discovered until the mid 1990s [56], [57]. Unlike NOS enzymes, the bacterial nitrate-reductase enzymes that convert nitrate to nitrite in the oral cavity function also function in the absence of oxygen. This means that this enterosalivary nitrate circulation becomes particularly important under hypoxic conditions when NOS activity becomes limited [58], [59], and nitrite-reducing pathways in mammalian tissues are still inhibited by the remaining oxygen levels [13].

Dietary nitrate supplementation initially increased the levels of salivary nitrate, nitrite and nitrosation products as expected – extra oral nitrate availability enables more bacterial nitrate to nitrite conversion, leading to enhanced systemic nitrite availability and formation of downstream nitrosation products [60]. However, it is surprising that hypoxic exposure did not significantly alter salivary nitrate or nitrite levels from sea level values. Moreover, we did not expect prolonged altitude exposure to narrow the difference in salivary RXNO concentrations between the two groups. The cause for this finding is not clear at present but this could be related to the duration of exposure as the trends suggest levels might still not have reached a plateau by day 5. Equally this could suggest that chronic hypoxic conditions might limit the maximum RXNO production rate, effectively placing a ceiling on the changes any dietary nitrate supplementation might induce.

### 4.4 Dietary nitrate and altitude illness

Our findings suggest that even though dietary nitrate supplementation at altitude results in increased biochemical nitrate availability, this does not improve acclimatization and/or reduce the incidence of AMS. Another hypobaric chamber study recently showed similar increases in E_NO_ after just a single dose of sodium nitrate. However, despite E_NO_ increasing by 24% at a simulated altitude of 4300 m, there was no demonstrable benefit in isometric exercise performance, forearm blood flow or tissue oxygenation [39]. Meanwhile another study, which recently exposed 20 healthy volunteers to 6 h of 12% oxygen in a normobaric chamber, actually showed an increase in AMS scores (particularly headache) with dietary nitrate, contrasting our findings [61]. Inhaled NO has previously been shown to reduce pulmonary arterial pressures both at sea level and at altitude, offering particular benefit to HAPE-susceptible patients who tend to have high pulmonary artery pressures [11], [12]. One explanation of these findings perhaps is that even a mild vasodilatory effect on the pulmonary vasculature confers benefits at altitude, particularly in people with naturally high pulmonary artery pressures. Meanwhile any cerebrovascular dilation in patients results in increased cerebrovascular flow and worsening of headache symptoms, particularly in those most susceptible to AMS.

### 4.5 Strengths and limitations

The Xtreme Alps study is one of the earliest double-blinded randomised placebo controlled trials of dietary nitrate supplementation at altitude that the authors are aware of, and also one of the larger studies to investigate the effects of nitrate metabolism at altitude. Nevertheless, absolute numbers remain rather moderate in each arm of the trial; we also had a number of missing data points due to subjects not being able to participate on some test days, usually due to altitude related illness. Consequently, confidence intervals remained wide in all reported measures so we cannot completely exclude a type-2 error, although a sensitivity analysis to correct for these missing values did not change these outcomes.

Since the Xtreme Alps study was conducted, other studies have emerged that also investigated how nitrate supplementation may affect performance at altitude, however most have used a simulated (chamber) altitude environment. In accordance with our findings, AMS incidence was shown to be similar to controls in cyclists taking dietary nitrate supplements exposed to short sessions of 11% oxygen in a normobaric chamber [62]. Similarly, short-term nitrate supplementation induced no significant changes in cognitive performance in healthy volunteers simulating hiking at altitude by walking on a treadmill in a normobaric chamber [63]. Curiously and contrary to our findings, all of these simulated altitude studies reported increases in SpO_2_ after nitrate supplementation [62], [63], [64], [65], [66]. The reason for this discrepancy is not clear. However, all of these results were in response to a very different physiological stimulus to the intervention presented here. Our subjects were exposed to hypobaric hypoxia gradually over a number of days; in chamber studies subjects tend to experience a much shorter duration and more rapidly induced period of normobaric hypoxia. For example, in one normobaric chamber study reporting an increase in cycling performance after just one dose of nitrate, subjects were decompressed to 15% oxygen in just 5 min [64]. Equally, the physiological response to normobaric and hypobaric hypoxia may not be the same, as suggested by a recent systematic review [67]. Currently Xtreme Alps remains the largest field study to date to explore the effects of dietary nitrate by taking serial measurements over a prolonged duration of hypobaric hypoxic exposure at high altitude.

Another study strength was the fact that the supplement we used to deliver nitrate (a natural beetroot/fruit juice blend) was almost identical in taste, appearance and composition to the placebo (the same mixture except that the beetroot/fruit juice was selectively reduced in nitrate). Ours is the only study we are aware of that also monitored nitrite and nitrate intake with the food.

As alluded to before, ascent occurred in two successive phases but inclement weather conditions meant that these ascent profiles could not be identical. However, statistical modeling has allowed us to control for these differences in ascent profile as much as possible, and trends between both ascent groups were similar. With each subject starting their beetroot juice supplement (containing either high nitrate 'treatment' or very low nitrate 'placebo') three days prior to each testing period, this meant we were unable to record baseline values at either sea level or at altitude. We were also unable to take a complete third 'treatment free' control group to altitude without reducing the power of the study due to the space available at 4559 m. However, the few measurements that were taken on the small number of treatment free laboratory team members all closely matched those of the placebo group suggesting that the low nitrate placebo supplement taken by this group did indeed behave as a placebo agent.

The majority of our subjects were young, non-smoking males with previous altitude experience, which is unsurprising given we primarily recruited from a mailing list of medical scientists and healthcare workers with an interest in high-altitude travel and hypoxia physiology. This sample remains representative of lowlanders who visit high altitude regions, as >83% of patients seen at the Himalayan Rescue Association's Everest Base Camp clinic over a ten-year period were male with an average age less than 40 [68]. Larger studies with a broader sample population might further strengthen this area of research in the future.

## 5 Conclusions

This study suggests that dietary nitrate supplementation is tolerable and safe at high altitude and robustly increases levels of E_NO_ as well as nitrate and nitrite concentrations in saliva, and nitrite concentrations in EBC. The association between EBC nitrite and Fe_NO_ levels suggests that exhaled NO may originate, at least in part, from nitrite in alveolar lining fluid. However, these increases in NO and related metabolites were not associated with any measureable changes in AMS, peripheral oxygen saturation or vital physiological parameters such as heart rate and blood pressure after five days of hypobaric hypoxic exposure. Future efforts should be directed at investigating whether dietary nitrate supplementation at altitude is associated with other physiological changes.

## Conflicts of interest

Together MF and HP designed, and HP produced and provided both the intervention and placebo supplement juice drinks for this study. MPWG serves on the medical advisory board of Sphere Medical Ltd and is a director of Oxygen Control Systems Ltd. He has received honoraria for speaking for and/or travel expenses from BOC Medical (Linde Group), Edwards Lifesciences and Cortex GmBH. MPWG leads the Xtreme- Everest Oxygen Research Consortium and the Fit-4-Surgery research collaboration. Some of this work was undertaken at University Southampton NHS Foundation Trust - University of Southampton NIHR Biomedical Research Centre. MPWG serves as the UK NIHR CRN national specialty group lead for Anaesthesia Perioperative Medicine and Pain and is an elected council member of the Royal College of Anaesthetists, an elected board member of the Faculty of Intensive Care Medicine, and President of the Critical Care Medicine Section of the Royal Society of Medicine. All other authors have nothing to declare.

## Funding

Xtreme Alps received charitable support from the Friends of University College Hospital NHS Foundation Trust as well as unrestricted research funding from Smiths Medical Ltd. and Deltex Medical Ltd. MP and MF acknowledge support from the Faculty of Medicine, University of Southampton. Analysis of the EBC and saliva samples was in partial fulfillment of the requirements for a MSc degree. Part of the work carried out at the University of Warwick was supported by funds from the Medical Research Council (G0701115) (Strategic Appointment Scheme, to MF). None of the funding bodies or the institutions the authors are affiliated with had any role in the study design; collection, analysis and interpretation of data; manuscript preparation, or decision to publish. AC is a National Institute for Health Research Academic Clinical Fellow.
